# Big data or bust: realizing the microbial genomics revolution

**DOI:** 10.1099/mgen.0.000046

**Published:** 2016-02-05

**Authors:** Sobia Raza, Leila Luheshi

**Affiliations:** PHG Foundation, Cambridge, UK

**Keywords:** data sharing, genomic data, pathogen genomics

## Abstract

Pathogen genomics has the potential to transform the clinical and public health management of infectious diseases through improved diagnosis, detection and tracking of antimicrobial resistance and outbreak control. However, the wide-ranging benefits of this technology can only fully be realized through the timely collation, integration and sharing of genomic and clinical/epidemiological metadata by all those involved in the delivery of genomic-informed services. As part of our review on bringing pathogen genomics into ‘health-service’ practice, we undertook extensive stakeholder consultation to examine the factors integral to achieving effective data sharing and integration. Infrastructure tailored to the needs of clinical users, as well as practical support and policies to facilitate the timely and responsible sharing of data with relevant health authorities and beyond, are all essential. We propose a tiered data sharing and integration model to maximize the immediate and longer term utility of microbial genomics in healthcare. Realizing this model at the scale and sophistication necessary to support national and international infection management services is not uncomplicated. Yet the establishment of a clear data strategy is paramount if failures in containing disease spread due to inadequate knowledge sharing are to be averted, and substantial progress made in tackling the dangers posed by infectious diseases.

## Impact Statement

Several recent articles have underlined the importance of ‘open-data’ for the development of microbial genomics research and its clinical utility. Achieving greater data sharing in practice is not straightforward. Herein we shift the discussions from ‘why’ onto ‘how’ to harness the potential of pathogen genomic data for healthcare. We describe the elements of an effective data management strategy, and consider how they could be arranged to deliver a proportionate system for aggregating and sharing pathogen genomic and associated clinical and epidemiological data. Our tiered-access model for data sharing could be adopted by any nation seeking to implement pathogen genomic services into its healthcare system. Realizing this model will demand the cooperation and collaboration of all those involved in the generation, delivery or distribution of pathogen genomics data and services.

## Introduction

Whole genome sequencing of pathogens is no longer the sole preserve of academic research and specialist centres. Falling costs and increasingly accessible forms of sequencing technologies have contributed to the growing use of genomics for both clinical and public health investigations. From the resolution of outbreaks in hospital wards (Köser *et al.*, 2012; Quick *et al.*, 2014), to national and international epidemiological investigations of disease sources (Gardy *et al.*, 2015; Loman *et al.*, 2013), genomics is helping to curtail the spread of infectious diseases and their devastating impact. Beyond disease surveillance, sequencing is enabling the earlier detection of emerging drug resistance in HIV-positive patients (Chabria *et al.*, 2014), and is poised to expedite the diagnostic management of *Mycobacterium tuberculosis* (Pankhurst *et al.*, 2016).

Despite the increasing number of elegant examples of genomics in action, many of the potential clinical benefits of this technology across the wide range of pathogens of importance to human health remain untapped. This is in large part due to the limited understanding of the clinical and epidemiological significance of genomic variation for many microbes. Theoretically the whole genome sequence of a pathogen can reveal its identity, virulence, drug resistance profile and relatedness to other pathogens. In practice these multifaceted applications will only surface as genomic data and associated ‘meta-data’ (phenotypic, clinical and epidemiological information) accrue and sequence variation can be confidently linked to specific phenotypic traits or clinical outcomes. Indeed, even the more immediate utility of pathogen genomics in monitoring and managing outbreaks is contingent on the ability to promptly combine and compare data and act upon this in a timely manner.

Collating, integrating and sharing data at scale and across the complex ecosystem of people and organizations involved in the management of infectious diseases does not come without huge technological, operational, political, ethical and regulatory challenges. As part of our review of what it will take to bring pathogen genomics into mainstream clinical and public health practice (Luheshi *et al.*, 2015), we consulted with multidisciplinary experts in the field to consider the factors most pertinent to the development of a data integration and management strategy. These discussions informed our vision for a data integration model which we believe can serve to maximize both the immediate and the future benefits of pathogen genomics if adopted by nations investing in this technology.

## Infrastructure: build it and they will come

A recent independent panel report on the global response to Ebola highlighted that the lack of reliable systems for sharing epidemiological, genomic and clinical data undermined effort to contain the outbreak (Moon *et al.*, 2015). Clearly, dedicated infrastructure or mechanisms for aggregating and accessing data are fundamental to any large-scale genomics enterprise. Too often vital pathogen genomics investigations during urgent public health pressures have relied on good will and ad hoc initiatives for data integration. For example, during their effort to determine the source of a hospital outbreak of *Salmonellaenterica* researchers resorted to the file-hosting service Dropbox to transfer genomic data to public health authorities who could then integrate this with their own surveillance data in order to trace the origin of the strain (Gardy *et al.*, 2015).

If genomic-based surveillance is to become routine and interventions to manage disease are to be more responsive to genomic information, then scalable and streamlined solutions for sharing and accessing data are essential. These mechanisms may well capitalize on the breadth and experience of existing large-scale sequence repositories – such as the National Center for Biotechnology Information database or the European Nucleotide Archive – an approach supported by the Global Microbial Identifier project. However, these publicly accessible systems were originally conceived to support research endeavours and so approaches to configure these resources to suit the needs of clinical and public health community – from controlled access to metadata, to speed and ease of data deposition and access – will all be necessary. Also needed are operational agreement and clear steer from national and international health authorities on the use of existing repositories for sharing and storing public health data.

## Incentives for sharing: carrots or sticks?

Beyond the availability of infrastructure, the ability and willingness of those generating pathogen genome data to share this information and do so in a useful format will dictate the success of genomics to improve infectious disease management. Whilst policies to incentivise the sharing of data generated in a research context have existed for some time, the use of pathogen genomics by clinical and public health investigators raises some distinct barriers to sharing. There will be huge variation in the location, approaches for generating and analysing data, and informatics capabilities of those deploying sequencing technologies for infection management. Data may arise from diagnostic operations in areas that are remote or underserved by healthcare (the case during the Ebola outbreak in West Africa), or from larger-scale higher-throughput public health surveillance facilities, such as the US Drug and Food Administration's GenomeTrakr network. Regardless of where the data are generated, failure to ensure their timely deposition and sharing, at the very least, with relevant public health authorities and/or authorized healthcare professionals will severely undermine the value of genomics to inform infection control. For this reason, we concluded that the sharing of data derived from clinical or public health investigations with the relevant health authorities should be viewed as mandatory.

Still, given the considerable variations in barriers and incentives to sharing, and the value of sharing data more widely, a mix of policies to facilitate and incentivize data sharing will be necessary. Actions could range from lessening the technical challenges to data deposition, reinforcing the value of data sharing by providing feedback on its use, to enforcing mandates and/or sanctions for those not complying. An extension of last of these is a suggestion of the Ebola response report which recommends a ‘naming and shaming’ approach for those countries that delay reporting outbreaks and sharing information (Moon *et al.*, 2015). Inevitably the challenges to data sharing are amplified when considering transnational data integration across different jurisdictions. Yet by developing strategies for data collation at a national level, individual countries can be better equipped to respond promptly to international surveillance efforts.

## Timing is everything

There are two levels of consideration regarding the timing of data sharing: sharing with public health authorities for the time-sensitive delivery of health protection services, and sharing beyond these frameworks into publicly accessible repositories to facilitate research development and improvements in services. Whilst the timing of sharing into the public domain is arguably more negotiable, from a health-service delivery perspective, the more rapid the deposition of data, the sooner outbreaks can be detected and the higher the chances of any public health intervention significantly limiting onward transmission. In essence, timely data sharing with those charged with health protection can save lives. Reports suggest that rapid information sharing combined with the mobilization of health workers for contact tracing and patient care helped to limit the Ebola outbreak in Senegal to one confirmed infection (Mirkovic *et al.*, 2014). The authors noted the importance of systems for strong cross-border communication in supporting disease containment. Yet even within borders a raft of political, regulatory and practical challenges impose barriers to timely data sharing. Current ‘research’-derived infrastructure for sharing data is not optimized for the speed and ease of use required by those delivering clinical applications. For example, other than challenges with data submission mechanisms, data must often be copied and transferred from the repository to local or cloud-based compute power in order to execute analyses, as these facilities are not collocated with the data repositories themselves. Other than mandates, facilitating the timely deposition of data will require bespoke infrastructure solutions and/or significant modifications to existing ‘research’ infrastructure to cater to the needs of the growing clinical user group.

## Transparency versus confidentiality: reaching a balance

Sharing data beyond health authority frameworks and into the public domain poses greater dilemmas. On the one hand, the swifter and greater the availability of data shared, the more effective the ability to drive innovation, expand services, develop therapeutics or even crowdsource analytical support (Rohde *et al.*, 2011). On the other hand, ‘open’ data sharing raises the risks of privacy breaches and harm to individuals or organizations identified as sources of infection. The political sensitivities surrounding the economic and reputational repercussions on industries, health systems and nations of correctly (or even incorrectly) being assigned ‘blame’ for a disease outbreak was exemplified during the Europe-wide outbreak of Shiga toxin-producing *Escherichia coli* outbreak in 2011 (Hyde, 2011). Reluctance to share data publicly can also stem from uncertainty surrounding data ownership and rights of end-users to benefits commercially, financially and academically from data generated by others. Inevitably strong political resolve both nationally and internationally will be vital to resolving these challenges. However, there are also complementary practical routes to balancing the risks and opportunities in ‘open-data’ sharing through the development and configuration of infrastructure. During our review we defined the principles and features of such a model which facilitates proportionate data sharing, supports data integration, and can ultimately serve the needs both of those delivering healthcare services and of wider user groups.

## A tiered model for data sharing

**Fig. 1.**
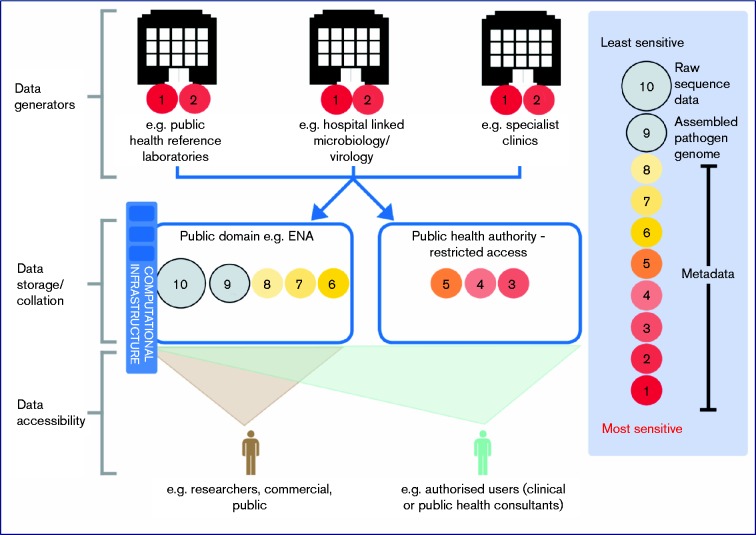
A tiered data sharing strategy for pathogen genomic and associated data. The size of circles (not to scale) are indicative of the relative data storage burden (computational disc space) of the different subsets of data.

Our consultation and research identified a tiered model for data sharing as the most optimal solution to maximizing the positive impact of pathogen genomics on health (Fig. 1). In this model access to the most sensitive levels of metadata would be limited to authorized healthcare professionals and/or those with justification for access, whilst placing all other genomic and less sensitive metadata, which pose minimal threats to patient or organizational confidentiality, in the public domain where they can be accessed by the widest possible range of researchers and public health and clinical practitioners. Interoperability of the ‘public’ access and ‘restricted’ access elements of the model would then enable authorized users to accurately link genomic data with all relevant sets of metadata.

For this tiered access model to operate, agreement on the types of metadata that should be subject to restricted access versus public release for each pathogen and application of genomics will need to be reached, as will the timing of data release into the public domain. Accepting that from a logistical and regulatory perspective this model will be more challenging and time consuming to develop than other alternatives, it is also the option most likely to deliver the improvement in our understanding of the basic biology of pathogens and in doing so drive innovation and the development of future pathogen genomic-informed services. Indeed many parallels can be drawn with the sharing of human (genomic) data in the context of improving and delivering safe and high-quality clinical genetic diagnostic services (Raza *et al.*, 2015). By capitalizing on the lessons and experience of ‘human’ genomics (Wright *et al.*, 2016), the microbial genomics community will be well placed to accelerate the clinical utility of sequencing technologies for infection management.

## Conclusion

Achieving a proportionate yet flexible system for collating, integrating and sharing data will demand political resolve, support and the cooperation of all those involved in pathogen genomics research and infectious disease management. This effort is not optional if lessons from recent and major outbreaks are to be seized, data mobilized and knowledge transformed into outcomes for patients and for public health.
